# Mitochondrial Elongation and ROS-Mediated Apoptosis in Prostate Cancer Cells under Therapy with Apalutamide and Complex I Inhibitor

**DOI:** 10.3390/ijms25136939

**Published:** 2024-06-25

**Authors:** Valentin Baumgartner, Dominik Schaer, Holger Moch, Souzan Salemi, Daniel Eberli

**Affiliations:** 1 Laboratory for Urologic Oncology and Stem Cell Therapy, Department of Urology, University Hospital Zurich, Wagistrasse 21, 8952 Schlieren, Switzerland; valentin.baumgartner@usz.ch (V.B.); daniel.eberli@usz.ch (D.E.); 2 Division of Internal Medicine, University Hospital Zurich, Wagistrasse 12, 8952 Schlieren, Switzerland; 3 Institute of Pathology and Molecular Pathology, University Hospital Zurich, Schmelzbergstrasse 12, 8091 Zurich, Switzerland

**Keywords:** prostate cancer, mitochondria, apalutamide, IACS-010759, oxidative stress

## Abstract

Metabolic reprogramming and mitochondrial dynamics are pivotal in prostate cancer (PCa) progression and treatment resistance, making them essential targets for therapeutic intervention. In this study, we investigated the effects of the androgen receptor antagonist apalutamide (ARN) and the mitochondrial electron transport chain complex I inhibitor IACS-010759 (IACS) on the mitochondrial network architecture and dynamics in PCa cells. Treatment with ARN and/or IACS induced significant changes in mitochondrial morphology, particularly elongation, in androgen-sensitive PCa cells. Additionally, ARN and IACS modulated the mitochondrial fission and fusion processes, indicating a convergence of metabolic and androgen-signaling pathways in shaping mitochondrial function. Notably, the combination treatment with ARN and IACS resulted in increased apoptotic cell death and mitochondrial oxidative stress selectively in the androgen-sensitive PCa cells. Our findings highlight the therapeutic potential of targeting mitochondrial metabolism in prostate cancer and emphasize the need for further mechanistic understanding to optimize treatment strategies and improve patient outcomes.

## 1. Introduction

Androgen deprivation therapy (ADT) has been a cornerstone in managing metastatic prostate cancer (PCa). Despite its efficacy initially, many patients eventually develop resistance to ADT, leading to disease progression. In the last decade, novel therapeutic strategies have emerged, including the use of androgen receptor (AR) antagonists such as apalutamide (ARN) [1], enzalutamide [2] and darolutamide [3]. However, continued application of these AR inhibitors induces drug resistance [4,5,6], necessitating alternative strategies and better combination therapies for a long-term clinical benefit in improving patient outcomes.

Beyond the canonical pathways targeted by anti-androgen therapies, emerging evidence suggests that metabolic reprogramming and stress response mechanisms play crucial roles in cancer progression and treatment resistance [7,8]. Among these mechanisms, alterations in oxidative phosphorylation (OXPHOS) and glycolysis have garnered significant attention for their contributions to tumor growth, survival, and adaptation to therapeutic stress [9]. Importantly, targeting specific metabolic properties of tumors has been proposed as a new strategy in overcoming drug resistance [10]. As the central hub of cellular metabolism, mitochondria are indispensable for energy production. Since many cancers have aberrant growth signals and energy needs, targeting the metabolic vulnerabilities of mitochondria could be a promising strategy [11]. Notably, PCa has specific metabolic dependencies. Normal prostatic epithelial cells are more reliant on glycolysis for energy production [12], but higher rates of mitochondrial metabolism activation have been observed with disease progression [13]. AR has a key regulatory role in rewiring energy metabolism, including the promotion of mitochondrial respiration and glycolysis [14,15]. However, the precise mechanisms through which AR induces metabolic reprogramming are not completely understood. Hence, investigating the role of AR in regulating mitochondrial signaling pathways could further explain the mechanisms of metabolic reprogramming and drug resistance.

Mitochondrial quality control is mainly governed by an important interplay between fission and fusion events, referred to as mitochondrial dynamics. These dynamic states of mitochondrial architectures have been linked to essential roles in responding to metabolic demands [16]. Hyperfragmentation has been reported in several cancers [17,18]. Importantly, prostate cancer stem cells and castration-resistant primary tumors showed an upregulated mitochondrial fission machinery [19]. Moreover, stress-induced mitochondrial fusion has been observed as a response to nutrient depletion [20]. This also suggests that cancer cells could bypass certain therapeutic drug treatments through this mitochondrial stress response by adapting their mitochondrial network.

Recently, we have demonstrated that an inhibitor of the mitochondrial electron transport chain complex I IACS-010759 (‘IACS’) acts synergistically with ARN in reducing PCa cell growth [21]. In this study, we investigated the effects of ARN and IACS on the mitochondrial network architecture and dynamics in PCa cells. Additionally, we explored the effect of androgen in regulating the mitochondrial network dynamics and metabolic modulations of respiratory pathways. Furthermore, we examined the impact of a combination therapy with ARN and IACS on regulating mitochondrial oxidative stress and apoptosis.

## 2. Results

### 2.1. ARN and IACS Modulate Mitochondrial Network Architecture and Morphology

**Figure 1:**
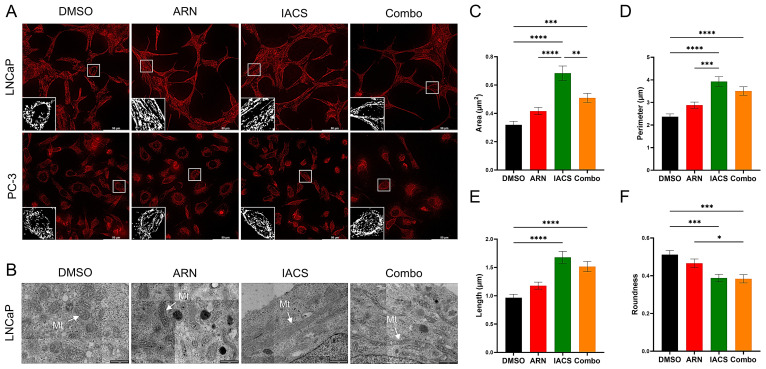
Mitochondrial network alterations in treated PCa cells. (**A**) Mitochondria were stained with TOM20 (red). Treatment with 25 µM apalutamide (ARN), 10 nM IACS-010759 (IACS) and Combo elongated mitochondria in LNCaP cells (top row). No morphological changes were observed in PC-3 cells upon drug treatment (bottom row). White boxed areas depict enlarged views of stained mitochondria. Scale bar: 50 µm. (**B**) TEM images visualize single mitochondria in LNCaP cells treated with DMSO, ARN, IACS and Combo. White arrows show representative mitochondria for each treatment condition. Scale bar: 1 µm. Quantification of *n* = 100 mitochondria for each treatment group with shape descriptors: area (**C**), perimeter (**D**), length (**E**) and roundness (**F**). Data are presented as means ± SEM. * *p* = 0.0458; ** *p* = 0.0030; *** *p* < 0.001; **** *p* < 0.0001. The uncropped TEM images are shown in Figure S1.

To study the impact of the drug treatment on the mitochondrial network dynamics, we visualized the mitochondria with TOM20 (Figure 1A). In androgen-dependent LNCaP cells, 72 h exposure to 25 µM ARN, 10 nM IACS, and the combination of both (ARN + IACS) prompted prominent shifts in network properties, notably towards a more tubular and filamentous morphology, in contrast to the vehicle control, where fragmented mitochondria predominated. In androgen-independent PC-3 cells, however, the same treatment did not seem to alter the mitochondrial network characteristics, as seen in LNCaP cells.

To investigate further that treatment with ARN and/or IACS serves as a modulator of mitochondrial dynamics, we analyzed the morphological features of treated LNCaP cells by transmission electron microscopy (TEM) (Figure 1B). For each condition, the shape of single mitochondria was examined (*n* = 100 per group) by measuring their area, perimeter, length and roundness (Figure 1C–F). The vehicle control mitochondria were observed as relatively round organelles with a mean length 0.97 ± 0.06 µm and an area of 0.32 µm^2^ ± 0.02. Treatment with IACS and Combo significantly elongated the mitochondria, as evaluated by an increased area (IACS: 0.68 µm^2^ ± 0.05, Combo: 0.51 µm^2^ ± 0.03), perimeter (IACS: 3.92 µm ± 0.22, Combo: 3.51 µm ± 0.19), and length (IACS: 1.68 µm ± 0.11, Combo: 1.52 µm ± 0.09), as well as decreased roundness (IACS: 0.39 ± 0.02, Combo: 0.38 ± 0.02). The ARN treatment alone yielded slightly lower effects compared to those observed with IACS and the Combo regimen, without significant differences compared to the control, with the ARN-treated cells exhibiting an area of 0.42 µm^2^ ± 0.03, a perimeter of 2.89 µm ± 0.13, a length of 1.18 µm ± 0.06, and a roundness of 0.47 ± 0.02.

### 2.2. Impact of Drug-Induced Mitochondrial Network Alterations on Fission and Fusion

**Figure 2:**
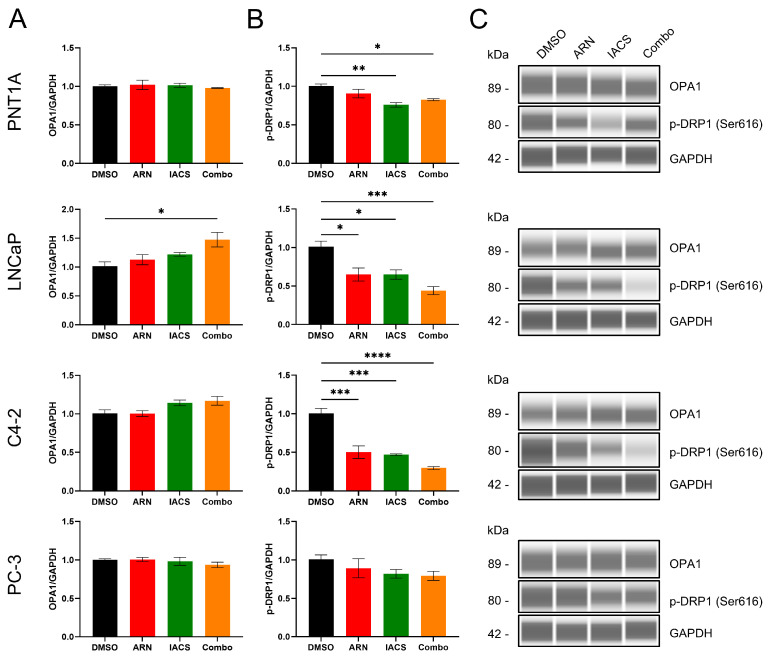
Treatment-induced changes in fission and fusion proteins. (**A**,**B**) Antibodies targeting OPA1 (**A**) and p-DRP1 (Ser616) (**B**) were quantified through automated Western blot analysis to determine changes in fusion and fission protein expression in PNT1A, LNCaP, C4-2 and PC-3 cells upon treatment with DMSO, ARN, IACS and Combo. The quantification of protein levels normalized to GAPDH is presented as means ± SEM (*n* = 3). * *p* < 0.05; ** *p* = 0.0034; *** *p* < 0.001; **** *p* < 0.0001. (**C**) Representative virtual immunoblots are shown for OPA1 and p-DRP1 (Ser616). Uncropped lanes are shown in Figures S2 and S3.

The potential treatment-induced, network-modulating effects were further assessed by measuring the expression levels of key proteins involved in mitochondrial dynamics: the mitochondrial fusion protein OPA1 (Figure 2A) and the fission protein p-DRP1 (Ser616) (Figure 2B) in different PCa cell lines. The mitochondrial fission protein was significantly reduced by 35% ± 8.4 (*p* = 0.0152) in LNCaP cells and by 49.7% ± 10 (*p* = 0.0004) in C4-2 cells upon ARN treatment compared to the control (Figure 2C). No significant changes were observed in p-DRP1 in PNT1A and PC-3 upon ARN treatment. Similarly, the IACS treatment reduced fission significantly in LNCaP by 35% ± 6 (*p* = 0.0152) and in C4-2 by 53% ± 1 (*p* = 0.0003). In PNT1A, IACS reduced fission by 24% ± 2.9 (*p* = 0.0034). The Combo treatment even further reduced the fission protein expression in LNCaP by 56% ± 5.1 (*p* = 0.0009) and in C4-2 by 70.3% ± 1.9 (*p* < 0.0001). The Combo treatment resulted in a reduction of 17.3% ± 1.5 in PNT1A (*p* = 0.0215). The IACS and Combo treatments had no significant effect on fission in PC-3 cells. The Combo treatment significantly increased OPA1 protein expression in LNCaP cells by 47.3% ± 12.6 (*p* = 0.0168). The C4-2 Combo-treated cells increased OPA1 expression by 17% ± 5.7, without reaching statistical significance. No changes in the fusion expression marker were observed in PNT1A and PC-3 (Figure 2C). Taken together, changes in fission and fusion markers were predominantly observed in the androgen-sensitive LNCaP and C4-2 cells. The significant reduction of p-DRP1 and moderate increase in OPA1 indicated the appearance of elongated mitochondria upon treatment with ARN and/or IACS.

### 2.3. Involvement of Androgen in Mitochondrial Functionality

**Figure 3:**
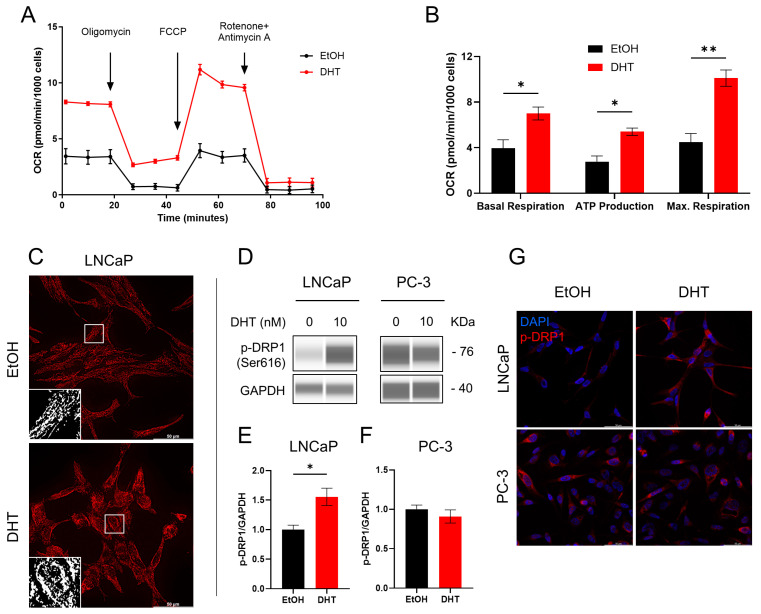
Androgen supplementation increases mitochondrial respiration and affects mitochondrial network. (**A**) Seahorse extracellular flux assay measured OCR in androgen-depleted (EtOH) and supplemented (DHT) LNCaP cells. Sequential addition of oligomycin, FCCP and rotenone + antimycin A were used to quantify basal respiration, ATP production and maximum respiration (**B**). (**C**) Cells were immunolabeled with TOM20 to visualize the mitochondrial network in EtOH-treated (top) and DHT-treated (bottom) LNCaP cells. (**D**) Protein levels with active fission marker p-DRP1 (Ser616) for LNCaP (left) and PC-3 cells (right), with or without DHT addition. GAPDH was used as a loading control. Quantification of p-DRP1 normalized to GAPDH for LNCaP (**E**) and PC-3 cells (**F**). (**G**) Immunostained LNCaP (top row) and PC-3 (bottom row) with p-DRP1 (red), with or without DHT addition. Nuclei were stained with DAPI (blue). Scale bar: 50 µm. Bar plots represent means ± SEM (*n* = 3). * *p* < 0.05; ** *p* = 0.0058. Uncropped Western blot lanes are shown in Figure S4.

To confirm the role of anti-androgen therapy on mitochondrial elongation, we starved LNCaP cells with CS-FBS to deplete the androgen levels and subsequently treated them with 10 nM DHT. Androgen supplementation increased the oxygen consumption rate (OCR) in DHT-treated cells compared to control (Figure 3A). The mitochondrial respiratory parameters of basal respiration, ATP production and maximal respiration were all significantly elevated upon DHT treatment (Figure 3B). Moreover, the androgen-depleted cells presented an elongated mitochondrial network in the TOM20 immunostained cells (Figure 3C). This effect could be reversed upon DHT addition, suggesting androgen-mediated mitochondrial reprogramming. Moreover, the fission marker p-DRP1 was significantly upregulated upon DHT addition in LNCaP cells but not in the androgen-independent PC-3 cells (Figure 3D–F). The LNCaP and PC-3 cells stained for p-DRP1 confirmed androgen-dependent upregulation of mitochondrial fission upon DHT treatment (Figure 3G).

### 2.4. Cytotoxic Effect of ARN and IACS in PCa Cell Lines

**Figure 4:**
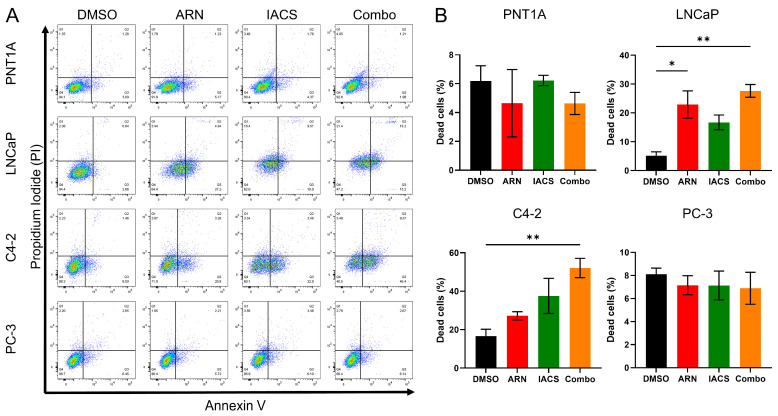
Assessment of apoptosis in PCa cells. (**A**) Representative flow cytometry dot plots of cells stained with Annexin V/PI. PCa cell lines PNT1A, LNCaP, C4-2 and PC-3 were treated for 72 h with DMSO, ARN, IACS and Combo. The sum of Annexin V (+)/PI (−) and Annexin V (+)/PI (+) cells in the lower right and upper right corner was used to quantify apoptotic cells. (**B**) Bar plots represent three independent experiments of measurements of apoptotic cells for PNT1A, LNCaP, C4-2 and PC-3. Bar plots are shown as means ± SEM. * *p* = 0.0179; ** *p* < 0.01.

Since we had evidence that androgen blockade, together with complex I inhibition, leads to alterations in the mitochondrial network, we wondered whether ARN and/or IACS have a cytotoxic effect on PCa cell lines. Figure 4A depicts a representative experiment illustrating Annexin V/PI-stained PCa cells following treatment with DMSO, ARN, IACS and Combo. A significant increase in the apoptotic cells in LNCaP compared to control was observed upon treatment with ARN (4.5-fold, *p* = 0.0179), IACS (3.3-fold) and Combo (5.4-fold, *p* = 0.0043). The Combo treatment led to significant apoptosis induction in C4-2 cells, by 3.1-fold (*p* = 0.0063) compared to the control cells. No cytotoxicity was observed in PNT1A and PC-3 cells with our treatment regimen (Figure 4B).

### 2.5. Treatment-Induced Mitochondrial Oxidative Stress in Androgen-Sensitive PCa Cells

**Figure 5:**
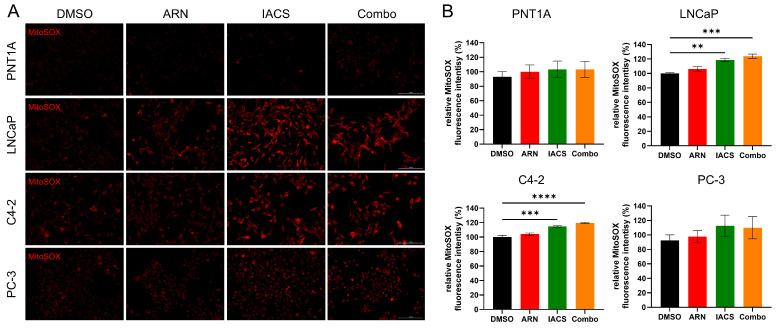
IACS and Combo increase mitochondrial ROS production in androgen-sensitive cells. (**A**) Oxidative stress in representative images of live cells labeled with MitoSOX (red) is shown. Scale bar: 200 µm. (**B**) Relative fluorescence intensity for MitoSOX was quantified for each cell line and treatment group (*n* = 3). Fluorescence intensity was normalized to DMSO control (100%). Bar plots represent means ± SEM. ** *p* < 0.01; *** *p* < 0.001; **** *p* < 0.0001.

Mitochondrial superoxide production was assessed in PCa cells upon treatment with ARN and/or IACS by image quantification of live cells. Mitochondrial ROS were examined in the MitoSOX-labeled cells by quantifying fluorescence intensity (Figure 5A,B). Fluorescence intensity was compared to DMSO control for all PCa cell lines (100%). IACS and Combo, but not ARN led to a significant increase in mitochondrial ROS in androgen-sensitive LNCaP and C4-2 cells. No significant changes were observed in PNT1A and PC-3 cells. Importantly, the measurement of general intracellular ROS showed no changes in fluorescence intensity upon treatment in LNCaP cells (Figure S5), suggesting treatment-induced mitochondria-specific modulation of superoxide production.

## 3. Discussion

Metabolic reprogramming is a major hallmark of cancer cells that enables rapid adaption to changes in energy needs for sustained cell survival and growth. Unlike Otto Warburg’s initial observation that tumor cells rely solely on aerobic glycolysis for energy production due to dysfunctional mitochondria [22], it is now well established that many tumors harbor functional mitochondria [23,24]. Indeed, PCa cells, in the early stages of the disease, have shown to activate their tricarboxylic acid (TCA) cycle due to reduced zinc levels [12]. Therefore, the reliance on OXPHOS is a promising therapeutic approach to target this metabolic vulnerability.

In this study, we show that treatment with ARN and/or IACS changes the mitochondrial network structure in androgen-dependent PCa cells towards elongated filamentous mitochondria. No changes in mitochondrial architecture were observed in AR-negative PC-3 cells with either ARN and IACS treatment. These observations pointed towards a role of androgen in modulating mitochondrial shape and, potentially, function or metabolism. Previous studies have reported that AR also localizes in the mitochondria through a mitochondrial localization sequence (MLS) and promotes the activity of mitochondrial respiration, as well as biogenesis [25,26]. Complex I blockade by IACS showed sensitivity in mitochondrial shape modulation exclusively in androgen-dependent cells. Androgen-sensitive PCa cells are highly dependent on OXPHOS for ATP production and therefore can be specifically targeted by complex I inhibition. On the other hand, PC-3 cells, which represent late-stage PCa, exhibit higher glycolytic activities [27]. Consequently, IACS treatment has shown no significant impact on these cells, underscoring the intricate metabolic rewiring mechanisms in PCa progression and drug resistance.

Mitochondrial fission and fusion processes are important in maintaining mitochondrial quality, biogenesis and removal of impaired mitochondria. Our results indicate that these dynamic states are affected by treatment with ARN and/or IACS, leading to a shift in the mitochondrial shape from fragmented towards elongated mitochondria, as indicated by reduced p-DRP1 (Ser616) and higher OPA1 protein expression levels. The quantitative analysis of the mitochondrial ultrastructure showed increased mitochondrial length and decreased roundness in LNCaP cells upon treatment. These results corroborate our previous observation of cells stained for mitochondrial marker TOM20 showing reduced fission and higher fusion, leading to an interconnected filamentous mitochondrial network. Accumulating evidence suggests that many cancer types exhibit an imbalance between mitochondrial fission and fusion, whereby excessive fission events seem to favor tumorgenicity [17,19,28,29,30]. Targeting the fission machinery through excessive mitochondrial segmentation has been shown to inhibit breast cancer cell migration through increased ROS levels [31]. In another study, targeting mitochondria with Gamitrinib-triphenylphosphonium (G-TPP), a mitochondrial matrix inhibitor, resulted in ROS-dependent elongated mitochondria and cell death [32]. These studies show that the modulation of mitochondrial dynamics seems to be highly context-dependent.

IACS treatment has been reported to induce a metabolic shift towards glycolysis in multiple cancer cell lines [21,33,34]. This compensation mechanism is characterized by low glucose levels, a known inducer of mitochondrial elongation [16,20,35]. This suggests a potential mechanism by which IACS treatment alters the mitochondrial morphology in prostate cancer cells through metabolic reprogramming. IACS has been evaluated in a recent phase I clinical trial and exhibited adverse effects in patients, particularly peripheral neuropathy [36]. It is therefore imperative to assess such adverse effects thoroughly in future preclinical studies exploring other mitochondria-targeting approaches.

Recently, we have shown that ARN and IACS treatment reduced the OXPHOS capacity in androgen-dependent PCa cells [21]. In this study, we contribute to those studies by demonstrating that androgen is involved in regulating complex pathways in mitochondrial respiration and network dynamics. Furthermore, we show that androgen supplementation via DHT increases respiratory parameters and induces a fragmented mitochondrial network through elevated p-DRP1 expression. These results suggest a pivotal role of androgen in regulating mitochondrial functionality. In line with these results are other studies that showed a mechanistic link of androgens in regulating DRP1 [37,38,39]. Additionally, our results indicated that anti-androgen treatment with ARN exhibits the reverse effect, inhibiting p-DRP1 and leading to an elongated network, further emphasizing the intricate role of androgens in modulating mitochondrial dynamics and function. Taken together, the elongated mitochondrial phenotype observed in response to the ARN and IACS treatments suggests a convergence of metabolic and androgen signaling pathways in shaping mitochondrial morphology and function in PCa cells. 

The significant elevation in mitochondrial ROS production in androgen-sensitive PCa cells following the combination treatment further supports the role of oxidative stress as a key mediator of apoptosis induction. Importantly, the lack of a significant increase in mitochondrial superoxide production and apoptotic cell death in benign PNT1A cells highlights the selectivity of the ARN and IACS treatments towards cancer cells, sparing normal cells from cytotoxic effects. The absence of significant changes in the general intracellular ROS in LNCaP cells suggests that oxidative stress induced by IACS and the combination treatment is mitochondria-specific. Consistent with the mitochondrial specificity of ROS induction seen in our results, a study found that in B-cell lymphoma, IACS treatment elevated the mitochondrial superoxide levels without a concurrent rise in H_2_O_2_ [34]. The same authors also showed that despite an increase in the antioxidant glutathione (GSH) levels, this compensation mechanism could not fully counteract ROS accumulation and likely overwhelmed the antioxidant defense system.

Additionally, the observed elongation of mitochondria in response to the ARN and IACS treatments could represent a stress response mechanism aimed at maintaining mitochondrial integrity and function. Mitochondrial elongation has been implicated as a protective response to cellular stress, serving to enhance mitochondrial function and mitigate apoptotic signaling pathways [16,20]. However, dysfunctional processes in removing damaged mitochondria, such as impaired mitophagy, could lead to the accumulation of ROS and the subsequent induction of apoptosis. Therefore, elucidating the mechanisms underlying mitochondrial dynamics and quality control processes in response to ARN and IACS treatments is crucial for understanding the therapeutic efficacy and potential resistance mechanisms in prostate cancer.

## 4. Materials and Methods

### 4.1. Pharmacological Compounds

Apalutamide (ARN) and IACS-010759 were both purchased from Selleck Chemicals (Houston, TX, USA). Stock solutions were prepared in DMSO and stored at −80 °C for ARN (50 mM) and IACS (10 mM). Hydrogen peroxide solution 30% (H_2_O_2_) was obtained from Sigma-Aldrich (St. Louis, MO, USA).

### 4.2. Cell Lines and Culture Conditions

PCa cell lines PNT1A (prostate epithelial cells), LNCaP (androgen-dependent) and PC-3 (androgen-independent) were all purchased from ATCC. C4-2 cells were a generous gift from Prof. George N. Thalmann (University Hospital Bern, Bern, Switzerland). The cells were grown in an RPMI medium (Thermo Fisher Scientific, Waltham, MA, USA), with the addition of 10% fetal bovine serum (FBS, Sigma-Aldrich, St. Louis, MO, USA) and 1% penicillin/streptomycin (P/S, obtained from Thermo Fisher Scientific, Waltham, MA, USA). Cells were maintained in a humidified incubator at 37 °C and 5% CO_2_.

### 4.3. DHT Treatment

To test androgen responsiveness, cells were starved for 24 h in 5% charcoal-stripped FBS (CS-FBS; Sigma Aldrich, St. Louis, MO, USA) and phenol red-free RPMI supplemented with 1% P/S. The starved cells were treated with 10 nM 5α-dihydrotestosterone (DHT, Sigma Aldrich, St. Louis, MO, USA). DHT was suspended in ethanol (EtOH). For immunocytochemistry and protein analysis, cells were treated with DHT for 3 days. For the Seahorse assay, cells were treated with DHT for 24 h.

### 4.4. Automated Western Blotting (WES)

Protein content was measured using the BCA assay (Thermo Fisher Scientific, Waltham, MA, USA). Capillary electrophoresis immunoassay (WES; ProteinSimple, automated Western blotting, San Jose, CA, USA) was performed to measure relative protein expression. Using a 12–230 kDa cartridge kit, the samples were prepared and analyzed according to the manufacturer’s protocol. The following primary antibodies were used: rabbit anti p-DRP1 (Ser616) (dynamin-related protein 1 phosphorylated at Ser616) (1:50, cell signaling, Danvers, MA, USA), rabbit anti OPA1 (optic atrophy 1) (1:50, Novus, Centinnial, CO, USA). Mouse anti GAPDH (1:100, Novus) served as a loading control. Immuno-detected proteins were quantitatively analyzed according to their molecular weight (MW) and chemiluminescent signal intensity (area). Compass software (ProteinSimple, V6.1.0) reported size- and charge-based analysis for our proteins of interest. Relative protein expression was normalized to GAPDH for each sample.

### 4.5. Seahorse Mito Stress Test

Mitochondrial respiratory parameters were measured by Seahorse extracellular flux assay (Agilent, Santa Clara, CA, USA). LNCaP cells were cultured for 24 h in CS-FBS. EtOH was added to a CS-FBS medium as a vehicle control. Next, 20,000 cells were seeded in XFe24 microplates (Agilent) and incubated for 24 h. The day after, the cells were treated with 10 nM DHT for an additional 24 h. On the day of the measurement, cells were washed 2× with Seahorse assay medium according to the manufacturer’s instructions: XF RPMI comprising 10 mM glucose, 1 mM pyruvate and 2 mM L-glutamine (all Agilent, Santa Clara, CA, USA). Mito Stress test reagents (Agilent) were prepared in an assay medium at 10× concentration and subsequently added into the loading ports: Oligomycin (15 µM), carbonyl cyanide 4-(trifluoromethoxy) phenylhydrazone (FCCP; 10 µM) and rotenone + antimycin A (10 µM). Oxygen respiration rate (OCR) was measured by Seahorse XFe analyzer in real-time with automatic sequential addition of mitochondrial stressors. Immediately after completion, nuclei were stained with Hoechst 33342 (Thermo Fisher Scientific). Images for stained nuclei were acquired using a Leica DMi8 microscope (Leica, Wetzlar, Germany) and quantified by ImageJ software (1.54 f NIH, Shah Alam, MD, USA) in order to normalize the OCR of measured cells.

### 4.6. Immunocytochemistry

Cells were cultured on 4-well chamber slides (Thermo Fisher Scientific, Waltham, MA, USA). Cells were fixed with ice-cold paraformaldehyde for 10 min. Washing with phosphate-buffered saline (PBS; Thermo Fisher Scientific, Waltham, MA, USA) was performed twice. Cells were then permeabilized with 0.2% Triton X-100 in PBS for 10 min, followed by 2 rounds of washing with PBS. Unspecific antibody binding was blocked by adding a blocking buffer (3% BSA + 1% normal goat serum in PBS) for 1 h at room temperature (RT). Rabbit anti TOM20 (outer membrane translocase complex, 1:200, Novus) or rabbit anti p-DRP1 (Ser616, 1:300, cell signaling) was added to the cells and incubated at 4 °C overnight. The next day, cells were incubated with secondary antibody goat anti-rabbit Cy3 IgG (1:700, Thermo Fisher Scientific) and DAPI (4′,6-diamidino-2-phenylindole, 1:200, Thermo Fisher Scientific) for 1 h, at RT. After washing with PBS, cells were mounted with coverslips using a DAKO mounting medium (Sigma Aldrich). Immunostained cells were imaged using a Leica DMi8 microscope.

### 4.7. Quantification of Mitochondrial Morphology by TEM

LNCaP cells were seeded on round coverslips in a 24-well plate and treated the next day with DMSO, 25 µM ARN, 10 nM IACS and a combination. At day 3 of treatment, the cell-culture medium was carefully removed and a fixation solution of 2.5% glutaraldehyde in 0.1 M Cacodylate buffer was added to cover the wells. Fixed cells were stored at 4 °C until further processing by the center for microscopy and image analysis, Zurich. Images were acquired by FEI Talos and evaluated by MAPS software (V3.21). For each treatment condition, 100 mitochondria were selected and quantified.

### 4.8. Measurement of Oxidative Stress

MitoSOX red mitochondrial superoxide indicator (Thermo Fisher Scientific, Waltham, MA, USA) was used for the measurement of cellular oxidative stress. Stock concentration of MitoSOX (5 mM) was prepared and 5 µM (1:1000 in culture medium) of the dye was added to cells in 96-well plates and incubated at 37 °C for 10 min in the dark. Hoechst 33342 (Thermo Fisher Scientific) was used to label the nuclei. Next, cells were washed with PBS and imaged by Cytation 5 (Agilent, Santa Clara, CA, USA). Fluorescence intensity was recorded (Ex/Em = 510/580 nm). For the measurement of general intracellular ROS, cells were stained with 5 µM of chloromethyl derivative 2′,7′-dichlorodihydrofluorescein diacetate (CM-H_2_DCFDA; Invitrogen, Waltham, MA, USA) according to the manufacturer’s instructions. Positive control cells were pre-treated for 1 h with 600 µM H_2_O_2_. Cells were stained in Hanks’ balanced salt solution (HBSS; Thermo Fisher Scientific, Waltham, MA, USA), containing the dye, for 30 min at 37 °C. Next, the cells were washed twice, returned to the growth medium and fluorescence intensity was recorded (Ex/Em = 485/528 nm) with a Cytation 5 microplate reader. 

### 4.9. Apoptosis Assay

Cytotoxicity was assessed in PCa cell lines by flow cytometry using the Annexin V-FITC apoptosis detection kit (Abcam, Cambridge, UK) according to the manufacturer’s protocol. Briefly, 200 µL of Annexin-binding buffer was added to the cell pellet in FACS tubes (BD) and placed on ice. An amount of 2 µL of Annexin V and 2 µL of propidium iodide (PI) were added to the cell suspension and incubated for 5 min at RT in the dark. Data were acquired using LSRFortessa (BD, San Jose, CA, USA) and analyzed by FlowJo (V10).

### 4.10. Statistical Analysis

Results were statistically analyzed using GraphPad Prism (GraphPad Software, Inc., La Jolla, CA, USA, version 9.5.1). Unpaired t-tests were used to compare differences in mitochondrial respiration parameters between control and DHT treated samples. Multiple groups were compared by one-way analysis of variance (ANOVA) with Bonferroni post-hoc tests. A significance level of *p* < 0.05 was considered statistically significant. Data are presented as means ± standard error of the mean (SEM).

## 5. Conclusions

In conclusion, our study reveals a complex relationship between androgen signaling, mitochondrial dynamics, and metabolic reprogramming in PCa. Treatment with ARN and/or IACS induces significant alterations in mitochondrial morphology, highlighting the potential of targeting mitochondrial metabolism as a therapeutic approach. However, it is important to consider the reported adverse effects associated with IACS. These adverse effects emphasize the need for careful monitoring and management when considering combination therapy. Further investigation into the mechanisms underlying these observations is warranted to optimize therapeutic strategies and improve treatment outcomes for PCa patients.

## Data Availability

All relevant data are included within this manuscript.

## Supplementary Materials

The following supporting information can be downloaded at: https://www.mdpi.com/article/10.3390/ijms25136939/s1.
