# Supplementary material for: Mitochondrial Elongation and ROS-Mediated Apoptosis in Prostate Cancer Cells under Therapy with Apalutamide and Complex I Inhibitor
Source: Int J Mol Sci. 2024 Jun 25;25(13):6939. doi: 10.3390/ijms25136939 (PMC11241170; doi:10.3390/ijms25136939)
Supplement: Supplementary file 1 [file ijms-25-06939-s001.zip › ijms-3032815-supplementary.pdf]

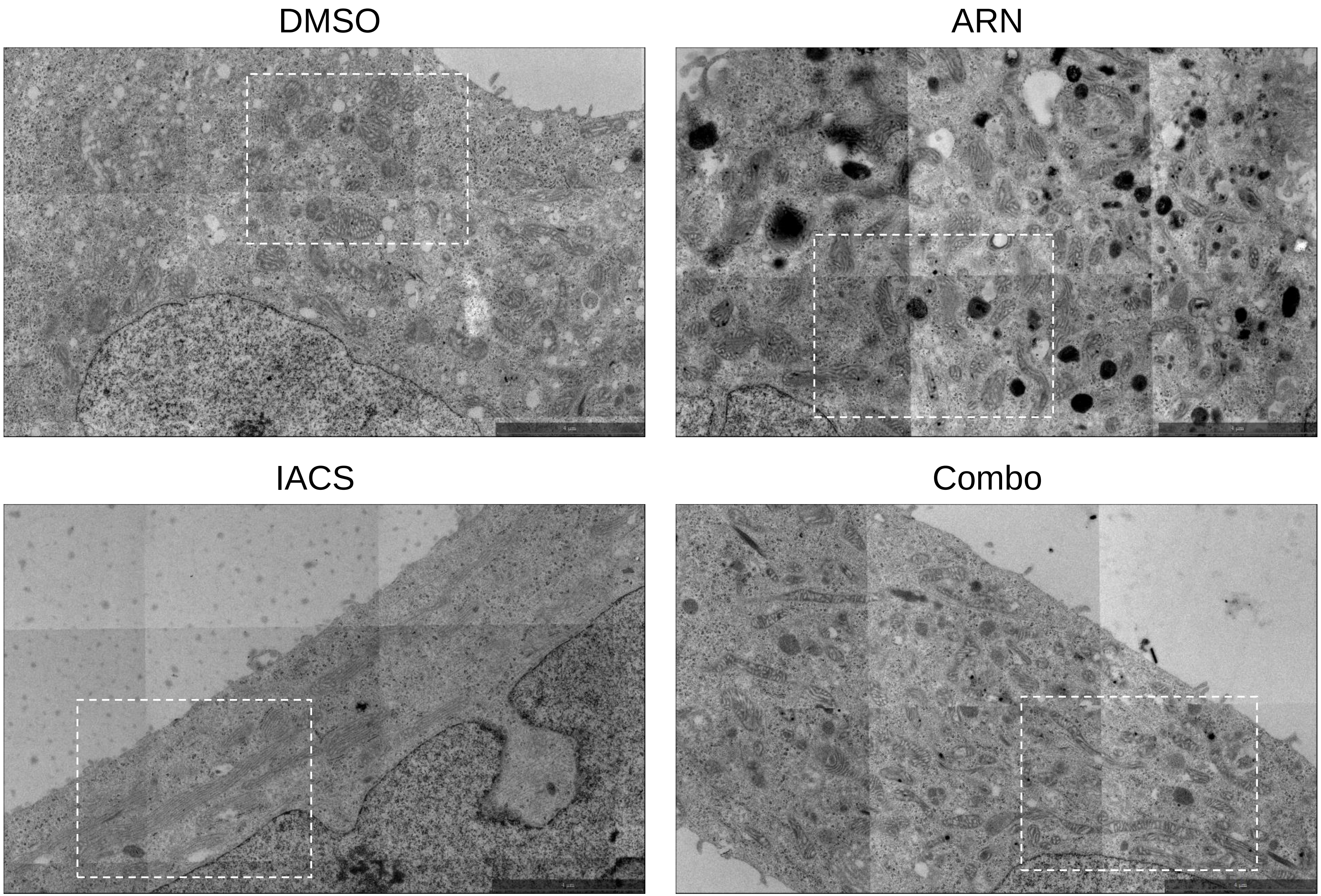

Figure S1. Uncropped TEM images showing mitochondrial ultrastructure. LNCaP cells were treated with either DMSO, 25 µM ARN, 10 nM IACS or a combination (Combo) for 72 hours. Dashed rectangles show representative images that were used in Figure 1B. Scale bar: 4 µm.

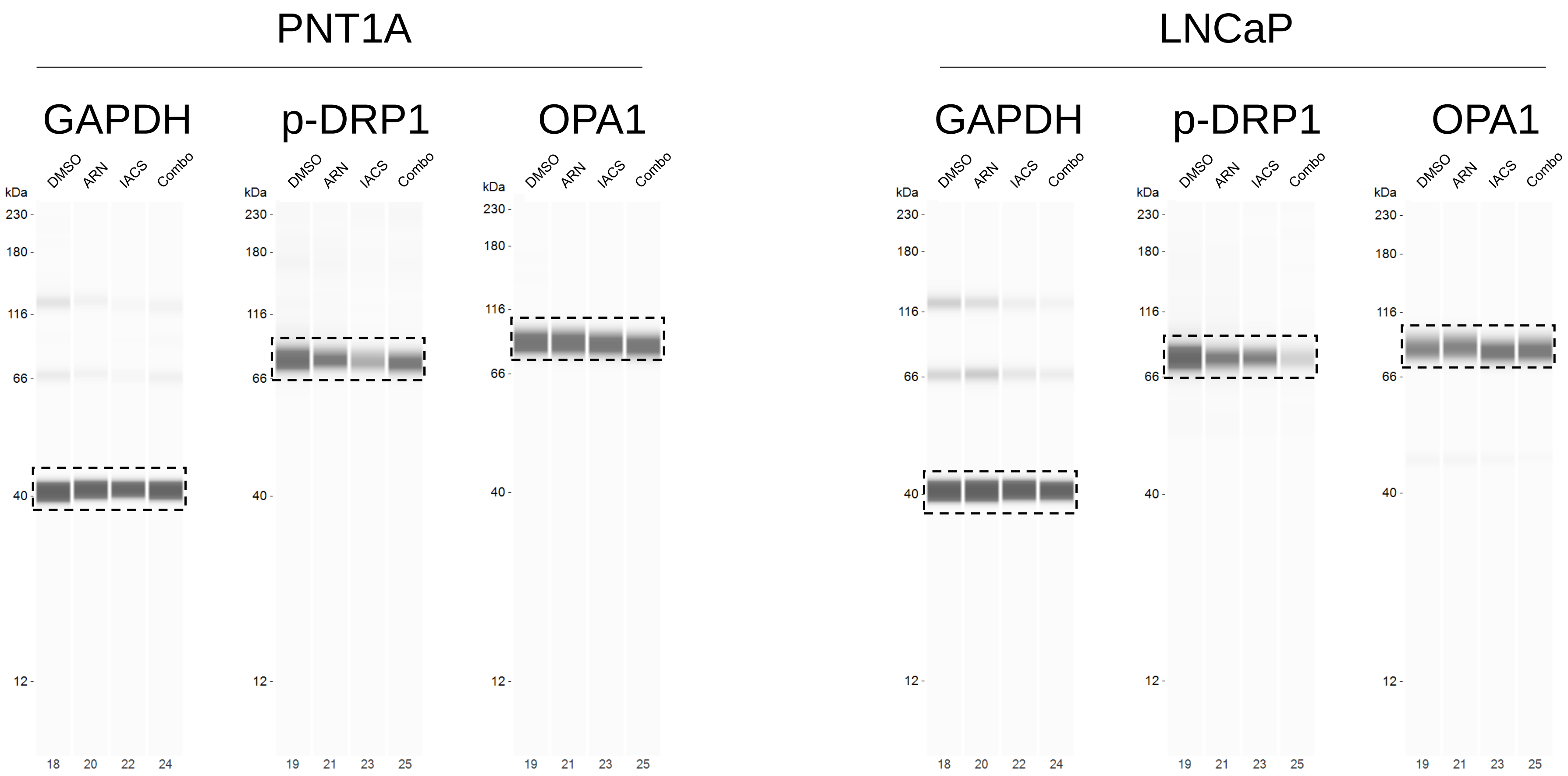

Figure S2. Uncropped images of western blots for PNT1A and LNCaP cells. Weight marker (kDa) and virtual lanes of GAPDH, p-DRP1, OPA1 are shown upon treatment with either DMSO, ARN, IACS or Combo for 72 hours. Dashed rectangles were used in Figure 2C.

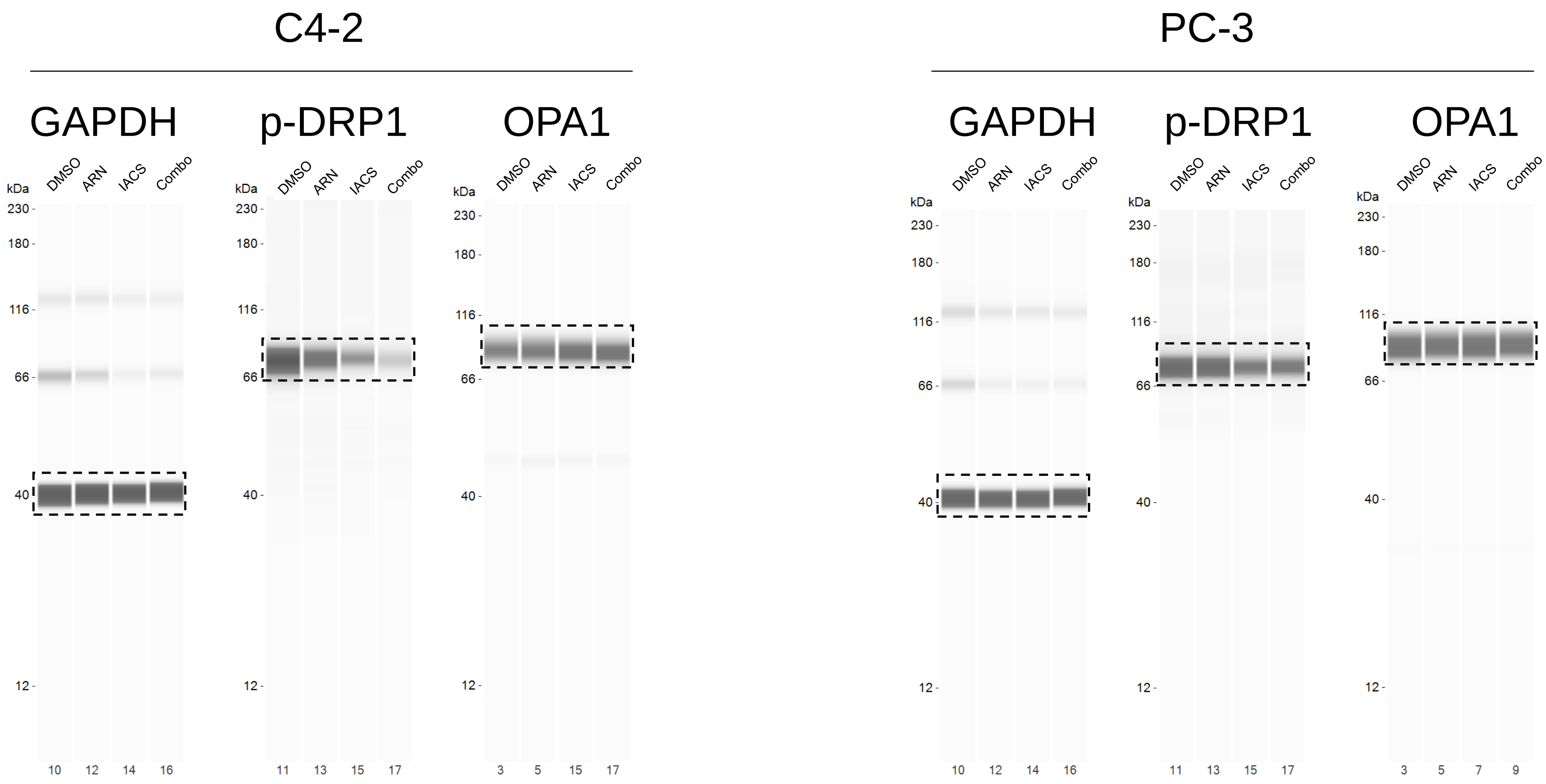

Figure S3. Uncropped images of western blots for C4-2 and PC-3 cells. Weight marker (kDa) and virtual lanes of GAPDH, p-DRP1, OPA1 are shown upon treatment with either DMSO, ARN, IACS or Combo for 72 hours. Dashed rectangles were used in Figure 2C.

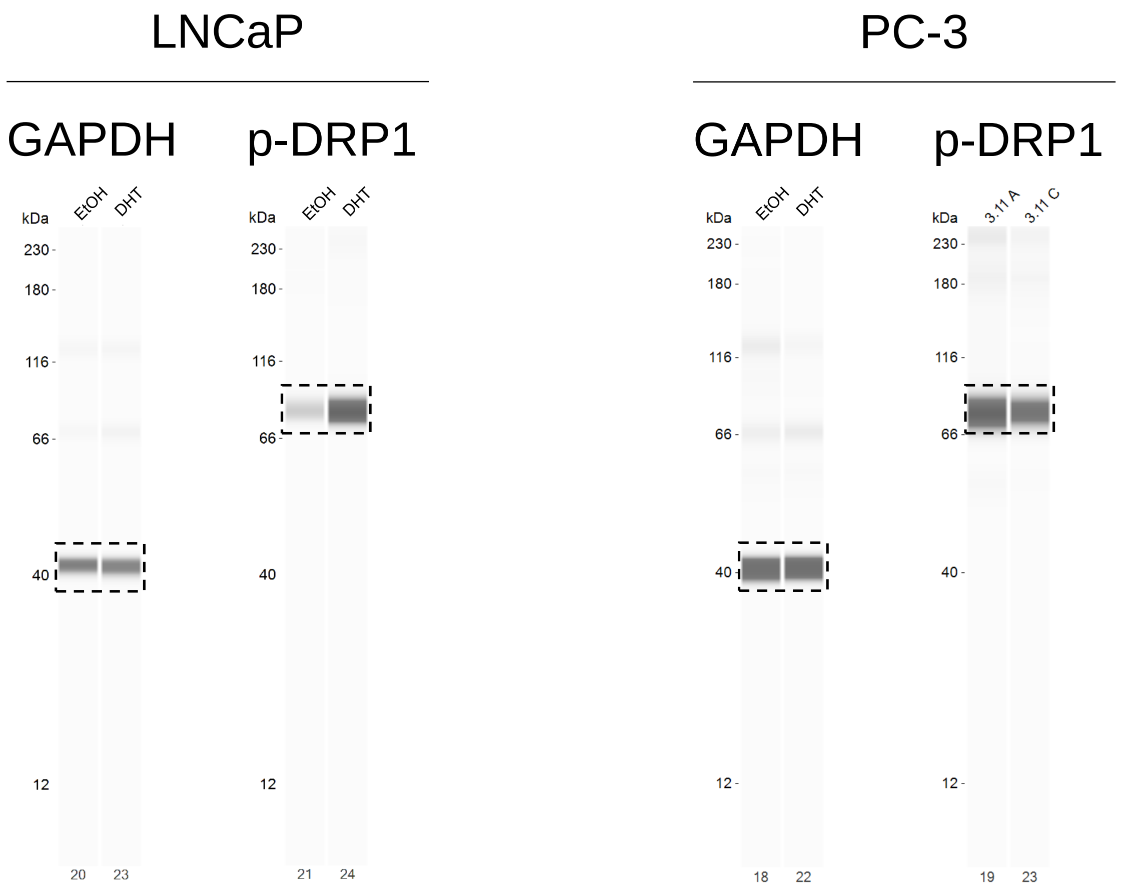

Figure S4. Uncropped images of western blots for LNCaP and PC-3 cells. Weight marker (kDa) and virtual lanes of GAPDH, p-DRP1 are shown for LNCaP and PC-3 cells upon treatment with either EtOH or 10 nM DHT for 72 hours. Dashed rectangles were used in Figure 3D.

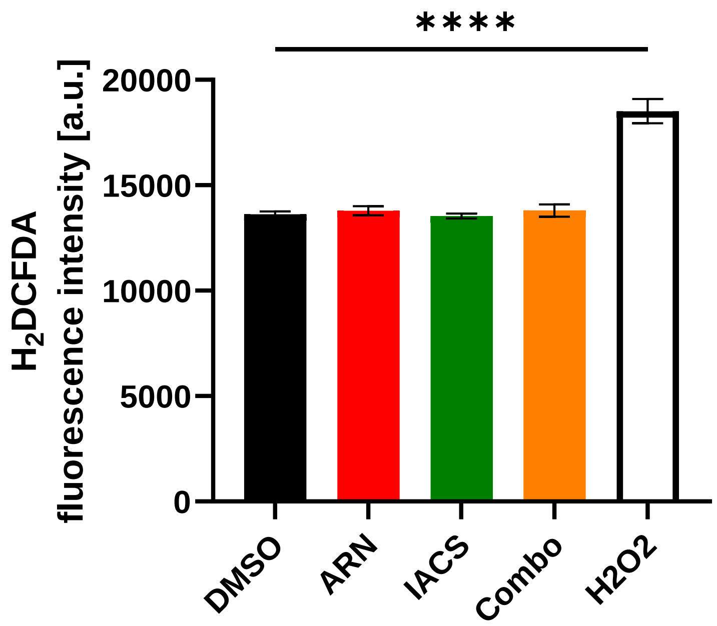

Figure S5. Fluorescence intensity measurements of general ROS indicator H_2DCFDA (*n*=3) in LNCaP cells. H_2O_2 served as a positive control. Bar plots represent means ± SEM. ****p<0.0001.
